# The efficacy of mouthwashes in reducing SARS-CoV-2 viral loads in human saliva: A systematic review

**DOI:** 10.1016/j.nmni.2022.101064

**Published:** 2022-12-12

**Authors:** Pardis Ziaeefar, Narjes Bostanghadiri, Parsa Yousefzadeh, Julian Gabbay, Amir Hashem Shahidi Bonjar, Mitra Ghazizadeh Ahsaie, Rosella Centis, Mohammad Sabeti, Giovanni Sotgiu, Giovanni Battista Migliori, Mohammad Javad Nasiri

**Affiliations:** a Department of Microbiology, School of Medicine, Shahid Beheshti University of Medical Science, Tehran, Iran; b Department of Microbiology, School of Medicine, Iran University of Medical Science, Tehran, Iran; c School of Dentistry, University of California, San Francisco, USA; d Clinician Scientist of Dental Materials and Restorative Dentistry, School of Dentistry, Shahid Beheshti University of Medical Sciences, Tehran, Iran; e Department of Oral and Maxillofacial Radiology, School of Dentistry, Shahid Beheshti University of Medical Sciences, Tehran, Iran; f Servizio di Epidemiologia Clinica delle Malattie Respiratorie, Istituti Clinici Scientifici Maugeri IRCCS, Tradate, Italy; g Clinical Epidemiology and Medical Statistics Unit, Department of Medicine, Surgery and Pharmacy, University of Sassari, Italy

**Keywords:** COVID-19, CT value, Mouth rinse, Mouthwashes, Salivary viral load, SARS-CoV-2

## Abstract

This systematic review aimed to evaluate existing randomized controlled trials (RCT) and cohort studies on the efficacy of mouthwashes in reducing SARS-CoV-2 viral loads in human saliva.

Searches with pertinent search terms were conducted in PubMed, MEDLINE, Scopus, and Web of Science databases for relevant records published up to Oct 15, 2022. Google Scholar and ProQuest were searched for grey literature. Manual searches were conducted as well for any pertinent articles. The protocol was prospectively registered at PROSPERO (CRD42022324894). Eligible studies were critically appraised for risk of bias and quality of evidence to assess the efficacy of mouthwash in reducing the SARS-CoV-2 viral load in human saliva.

Eleven studies were included. The effect on viral load using various types of mouthwash was observed, including chlorhexidine (CHX), povidone-iodine (PI), cetylpyridinium chloride (CPC), hydrogen peroxide (HP), ß-cyclodextrin-citrox mouthwash (CDCM), and Hypochlorous acid (HCIO). Eight articles discussed CHX use. Five were found to be significant and three did not show any significant decrease in viral loads. Eight studies reviewed the use of PI, with five articles identifying a significant decrease in viral load, and three not showing a significant decrease in viral load. HP was reviewed in four studies, two studies identified significant viral load reductions, and two did not. CPC was reviewed in four studies, two of which identified significant viral load reductions, and two did not. CDCM was reviewed in one article which found a significant decrease in viral load reduction. Also, HCIO which was evaluated in one study indicated no significant difference in CT value.

The current systematic review indicates that based on these eleven studies, mouthwashes are effective at reducing the SARS-CoV-2 viral load in human saliva. However, further studies should be performed on larger populations with different mouthwashes. The overall quality of evidence was high.

## 1 Introduction

Severe acute respiratory syndrome coronavirus 2 (SARS-CoV-2) was first discovered in December 2019 in Wuhan (China) followed by a rapid worldwide spread in a short duration of time. Multiple public health strategies such as mandated mask-wearing, medications, and vaccinations have been recommended to control the epidemiology of the pandemic. Medications and vaccinations are designed to target the cell structure of the coronavirus, which helps reduce virulence [1].

Cell entry mechanisms of SARS-CoV-2 are based on the binding of the virus spike protein and the human angiotensin-converting enzyme 2 (ACE2) receptor [2]. High expression of ACE2 receptors in oral and nasopharynx epithelium makes the oral cavity a vulnerable anatomical target [3]. Using mouthwashes that are low-cost and feasible for all individuals to reduce the risk of COVID-19 infection as a prevention strategy is noteworthy [4]. In addition, dental professionals and their patients could be at risk of transmission and infection following the aerosol produced by dental instruments [5]. Mouthwashes might reduce the individual risk.

Several studies evaluated the role played by mouthwashes in the reduction of the risk of SARS-CoV-2 infection; however, conflicting data on their efficacy were published. The present study aims to systematically review the efficacy of mouthwashes in reducing SARS-CoV-2 viral loads in human saliva.

## 2 Methods

The Preferred Reporting Items for Systematic Reviews were adopted to describe the methodology and results [6] (PROSPERO 2022 CRD42022324894) of the present systematic review.

***Population:*** Patients infected by SARS-CoV-2.

***Interventions:*** Different mouthwashes, including chlorhexidine (CHX), povidone-iodine (PI), cetylpyridinium chloride (CPC), hydrogen peroxide (HP), ß-cyclodextrin-citrox mouthwash (CDCM), and Hypochlorous acid (HCIO).

***Comparisons:*** Distilled water and placebo.

***Outcomes:*** Saliva viral loads.

***Study designs:*** Randomized controlled clinical trials (RCTs) and observational cohort studies.

### 2.1 Search strategy

PubMed/MEDLINE, Scopus, Web of Science, Google Scholar, and ProQuest databases were searched for studies reporting on the efficacy of mouthwashes against SARS-CoV-2, published up to Oct 15, 2022.

The following search terms were used: (COVID-19) OR (Coronavirus Disease 2019) OR (SARS-CoV-2) AND (mouthwashes) OR (mouth rinse) OR (mouth bath) OR (mouth wash) OR (chlorhexidine) OR (hydrogen peroxide) OR (hydroperoxide) OR (saline solution) OR (oral rinse) OR (povidone-iodine) OR (chloride) OR (cetylpyridinium) OR (oral hygiene). Only studies written in English were selected.

All records were imported into the bibliographic software EndNote X7 (Thomson Reuters, Toronto, ON, Canada).

### 2.2 Study selection

The records found through database searching were merged, and duplicates were removed. Two reviewers (P.Z. and P.Y.) independently screened records by title/abstract and full text to exclude those unrelated to the study topic. Studies meeting the following criteria were included: (1) inclusion of patients diagnosed with SARS-CoV-2 infection; (2) inclusion of patients exposed to mouthwashes; and (3) description of the outcome of saliva viral load. Calibration between reviewers was performed by a joint evaluation of the first 15 consecutive articles. Disagreements were resolved by consulting a third reviewer (M.J.N.). Reasons for exclusion at the full-text stage were recorded. Conference abstracts, editorials, reviews, study protocols, in-vitro studies, and molecular or experimental studies on animal models were excluded.

### 2.3 Data extraction

The following data were extracted and placed into an excel spreadsheet (Microsoft, Redmond, WA): First author's name; year of publication; study duration; type of study; the country where the study was conducted; the number of patients with SARS-CoV-2 infection; patient age; treatment protocols; demographics (i.e., age, sex, nationality); and treatment outcome.

### 2.4 Risk of bias assessment

Two reviewers (P.Z. and P.Y.) independently used two tools to assess study quality: The Newcastle-Ottawa Scale (NOS) for observational studies, and 2) the Cochrane risk of bias tool for experimental studies [7,8]. The Cochrane risk of bias tool evaluated the studies across seven different domains: random sequence generalization, allocation concealment, blinding of participants and personnel, blinding of outcome assessment, incomplete outcome data, selective reporting, and other bias. A study was determined to be “low risk” if all categories were rendered as low risk. If one domain was categorized as possessing an unclear risk of bias, the paper was classified as having “some risk of bias.” If the study had three or more domains that had an unclear risk or had one category considered high risk, the study was classified as “high risk.” The NOS scale was used to evaluate the risk of bias in observational studies across three domains: (1) selection of participants, (2) comparability, and (3) outcomes. A study can be awarded a maximum of one point for each numbered item within the selection of participants and outcome categories. A maximum of two-point can be given for comparability. Scores of 0–3, 4–6, and 7–9 were assigned for the low, moderate, and high-quality studies, respectively. Disagreements were resolved by consultation with a third reviewer (M.J.N.).

## 3 Results

### 3.1 Search results

**Fig. 1:**
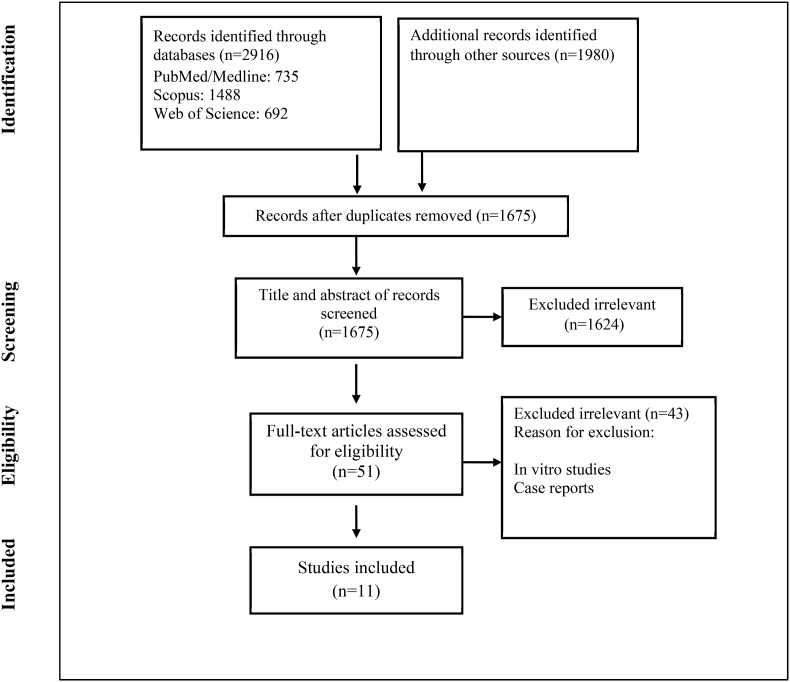
Flow chart of study selection for inclusion in the systematic review.

A total of 2,915 records were found. After removing duplicates, 1,675 titles and abstracts were screened (Fig. 1). Of these, 51 articles were selected for a full-text review and 11 met the inclusion criteria: Two observational and nine experimental studies [[9], [10], [11], [12], [13], [14], [15], [16], [17], [18], [19]].

### 3.2 Study Characteristics

#### 3.2.1 Population

The eleven studies included a total of 1020 patients ranging from 18 to 90 years old (Table 1). All participants were COVID-19 patients whose diagnosis was confirmed via the detection of SARS-CoV-2 by reverse transcriptase-polymerase chain reaction (RT-PCR). Exclusion criteria consisted of patients with allergies to any of the mouthwashes, nasogastric or endotracheal tubes, thyroid disease, renal failure, developmental/cognitive disability, severe acute or chronic medical or psychiatric condition, pregnancy, current radioactive iodine treatment, or antiseptic mouthwash consumption 48 hours before the start of the study.

**Table 1:** Characteristics of included studies.

First author	Year	Location	Type of study	Participants age range	Total number of participants	Intervention	Control solution	Duration of intervention	Intervention (no.)	Control (no.)	Definition of outcome	Outcomes
Huang [13]	2020	USA	Observational	23-89	294	0.12%CHX Oral rinse	NR	4 days	66	55	Salivary viral load based on CT-value using RT-PCR	Significant difference
						0.12%CHX Oral rinse posterior oropharyngeal spray			93	80		
Seneviratne [14]	2020	Singapore	Observational	NR	16	0.5%PI	Distilled water	1 day	4	2	Salivary viral load based on CT-value using RT-PCR	Significant difference 6 h post-rinsing
						0.12%CHX			6			No significant difference
						0.075%CPC			4			Significant difference 5 min and 6 h post-rinsing
Elzein [15]	2020	Lebanon	Experimental	17-85	61	0.2%CHX	Distilled water	1 day	25	9	Salivary viral load based on CT-value using RT-PCR	Significant difference 5 min post-rinsing
						1%PI			27			Significant difference 5 min post-rinsing
de Paula Eduardo [10]	2020	Brazil	Experimental	≥18	43	0.075%CPC+0.28%Zinc lactate	Distilled water	1 day	7	9	Salivary viral load based on CT-value using RT-PCR	Significant difference immediately after rinsing
						1.5%HP			7			Significant difference immediately after rinsing, 30 min and 60 min post-rinsing
						0.12%CHX			8			Significant difference 30 min and 60 min after rinsing
						1.5%HP+0.12%CHX			12			Significant difference immediately after rinsing
Ferrer [11]	2020	Spain	Experimental	>18	58	2%PI	Distilled water	1 day	9	12	Salivary viral load based on CT-value using RT-PCR	No significant difference
						1%HP			14			No significant difference
						0.07%CPC			11			No significant difference
						0.12%CHX			12			No significant difference
Chaudhary [12]	NR(2021)	USA	Experimental	24-82	40	Normal saline	NR	1 day	NR	NR	Salivary viral load based on CT-value using RT-PCR	Significant difference between baseline viral load and viral load at 15 min and 45 min post-rinsing
						1%HP						
						0.12%CHX						
						0.5%PI						
Arefin [16]	2020	Bangladesh	Experimental	≥18	189	0.4, 0.5, 0.6% PI (NI) 0.5, 0.6% PI (NS)	Distilled water	1 day	162	27	Salivary viral load based on CT-value using RT-PCR	Significant difference
Carrouel [9]	2020	France	Experimental	18-85	154	CDCM	Placebo	7 days	88	88	Salivary viral load based on CT-value using RT-PCR	Significant difference 4 h post-rinsing No significant difference at day 7
Natto [17]	2022	Saudi Arabia	Experimental	≥18	60	CHX (mouth rinse)	Saline	1 day	45	15	Salivary viral load based on CT-value using RT-PCR	No significant difference between all groups at two times Significant reduction in viral load of all four groups Significant reduction of both salivary load and CT value in PI
						CHX (lozenges)						
						PI						
Sánchez Barrueco [18]	2022	Spain	Experimental	25-90	44	2% PI	Distilled water	1 day	9	Salivary viral load		No significant difference
						1% HP			6			
						0.07% CPC			10			
						0.12% CHX			9			
Sevinç Gül [19]	2022	Turkey	Experimental	20-83	61	0.02% HCIO	0.9% Saline	1 day	20	20	Salivary viral load based on CT-value using RT-PCR	No significant difference
						0.5 % PI			21			

CHX; Chlorhexidine, PI; Povidone Iodine, HP; Hydrogen peroxide, CPC; Cetylpyridinium Chloride, ß-cyclodextrin citrox mouthwash, HCIO; Hypochlorous acid, NI; Nasal irrigation, NS; Nasal spray, NR; not reported.

#### 3.2.2 Intervention

The following mouthwashes were prescribed: CHX in eight studies with the dosage of 0.12, and 0.2% [[10], [11], [12], [13], [14], [15],^17^,^18^], PI with the dosage of 0.4, 0.5, 0.6, 1, and 2% in eight studies [11,12,[14], [15], [16], [17], [18], [19]], the dose of 0.07 % of CPC in four studies [10,11,14,18], HP with the dosage of 1, and 1.5 % in four studies [[10], [11], [12],^18^], CDCM in one study [9], and 0.02% HCIO in one study [19]. Mouth rinses in the intervention groups were compared with distilled water as the control groups for nine of the studies [[10], [11], [12],[14], [15], [16], [17], [18], [19]]. In the study of Carrouel et al., the control group used a placebo as mouthwash [9]. That is to say, the same mouthwash that the test group but without the active molecules (citrox and b-cyclodextrin). In the study of Huang et al., the control group didn't use mouthwash [13].

#### 3.2.3 Outcome assessment

The outcome was measured through RT-PCR: % reduction in viral load was the target. Studies evaluated salivary viral load clearance with CT value changes [9,10,12,14,15,[17], [18], [19]] and/or RT-PCR [14], quantitative RT-PCR [[9], [10], [11], [12],^18^], qualitative RT-PCR [16], and real-time RT-PCR [13,15,17,19].

### 3.3 Risk of bias

**Fig. 2:**
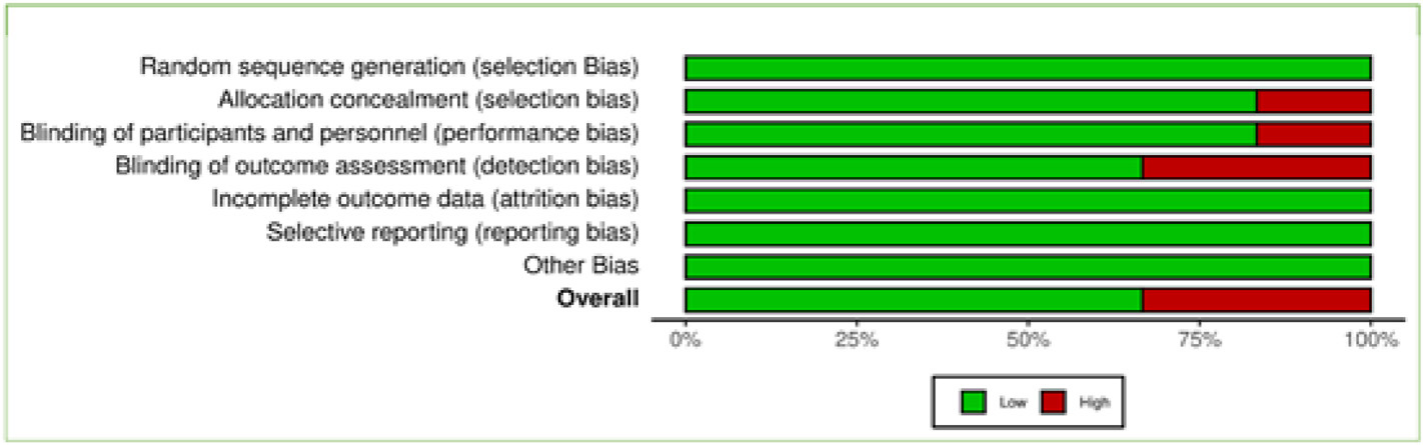
Review of the author's assessment of the risk of bias domains for each experimental study, displayed as percentages across included studies.

The mean (standard deviation [SD]) NOS score was 8.0 (0.6), which is suggestive of high methodological quality and a low risk of bias (Table 2). Four studies had a low risk of bias in all seven domains [9,11,12,15] (Table 3 and Fig. 2). One study has a high risk of bias in cases of allocation concealment, blinding of participants, and blinding of the outcome; and a low risk of bias in all other domains [16]. De Paula Eduardo et al. had a high risk of bias in the blinding of outcome assessments, and a low risk of bias in all other categories [10].

**Table 2:** Quality assessment of the observational studies included in the meta-analysis (The NOS tool).

Author	Selection				Comparability		Outcome			
	Representativeness of exposed cohort	Selection of non-exposed cohort	Ascertainment of exposure	Demonstration that outcome of interest was not present at start of study	Adjust for the most important risk factors	Adjust for other risk factors	Assessment of outcome	Follow-up length	Loss to follow-up Rate	Total quality score
Huang et al. [13]	1	1	1	1	1	0	1	1	1	8
Seneviratne et al. [14]	1	1	1	1	1	0	1	1	1	8

**Table 3:** Quality assessment of the experimental studies included in the meta-analysis (the Cochrane tool).

Author	Random sequence generation	Allocation concealment	Blinding of participants andpersonnel	Blinding of outcome assessment	Incomplete outcome data	Selective reporting	Other bias
Elzein et al. [15]	Low risk	Low risk	Low risk	Low risk	Low risk	Low risk	Low risk
de Paula Eduardo et al. [10]	Low risk	Low risk	Low risk	High risk	Low risk	Low risk	Low risk
Arefin et al. [16]	Low risk	High risk	High risk	High risk	Low risk	Low risk	Low risk
Carrouel et al. [9]	Low risk	Low risk	Low risk	Low risk	Low risk	Low risk	Low risk
Ferrer et al. [11]	Low risk	Low risk	Low risk	Low risk	Low risk	Low risk	Low risk
Chaudhary et al. [12]	Low risk	Low risk	Low risk	Low risk	Low risk	Low risk	Low risk
Natto et al. [17]	Low risk	Low risk	Low risk	Low risk	Low risk	Low risk	Low risk
Sánchez Barrueco wt al [18].	Low risk	Low risk	Low risk	Low risk	Low risk	Low risk	Low risk
Sevinç Gül et al. [19]	Low risk	Low risk	Low risk	Low risk	Low risk	Low risk	Low risk

#### 3.3.1 Chlorhexidine

Eight studies evaluated the efficacy of CHX [[10], [11], [12], [13], [14], [15],^17^,^18^]. Four examined 0.12% CHX mouth rinses and measured viral load reduction at 5 and 60 minutes. The studies found a significant decrease in viral load at both times [10,12,13,15]. Three studies observed viral load four days after continuous daily use of CHX and also found significant viral load reduction [10,13,15]. Rinsing with HP mouthwash followed by CHX mouthwash reduced viral load immediately after consumption [10]. Significant viral load clearance was seen following four days of usage of CHX oral rinse with a posterior oropharyngeal spray (86%) via swabbing the oropharynx to test the presence of SARS-CoV-2 by rRT-PCR. The combination of CHX oral rinse with a posterior oropharyngeal spray in addition to the second study group, the aforementioned combination was used in 15 healthcare workers as a preventive strategy in addition to hand sanitizing, mask-wearing, and social distancing. None of the healthcare workers developed COVID-19 infection during the study time, whilst nearly 50% of all healthcare workers in their respective hospitals developed infection [13]. Also, in the study conducted by Natto et al., both CHX mouthrinse and lozenges were administrated and significantly reduced the viral load [17]. However, three studies indicated no significant difference using the CHX mouth rinses [11,14,18]. (Table 1).

#### 3.3.2 Povidone-iodine

Eight studies surveyed the efficacy of PI on the SARS-CoV-2 viral load [11,12,[14], [15], [16], [17], [18], [19]]. Five studies confirmed its efficacy as follows [12,[14], [15], [16], [17]]; 5 minutes post-rinsing of 1% PI, 15 and 45 minutes, and 6 hours after 0.5% PI. Similarly, different concentrations of PI in forms of nasal irrigation (NI) and nasal spray (NS) indicated showed viral load clearance; however, a significant viral load reduction of 0.5% PI NI compared to 0.5% PI NS was found. The most incident adverse event was nasal irritation [16]. Three studies found that 0.5% [19] and 2% PI [11,18] consumption did not have any significant efficacy in viral load reduction.

#### 3.3.3 Hydrogen peroxide

Four studies evaluated the effectiveness of HP [[10], [11], [12],^18^]. 1.5% HP significantly reduced SARS-CoV-2 viral load immediately, after 30 and 60 minutes [10]. HP at a concentration of 1%, evaluated with 15 and 45-minute post-rinsing measurements, significantly reduced the SARS-CoV-2 load [12]. Two studies using 1% HP as a mouth rinse indicated no significant efficacy on viral load clearance [11,18].

#### 3.3.4 Cetylpyridinium chloride

A total of four studies evaluated CPC efficacy [10,11,14,18]: two reported the effectiveness shortly after administration and showed a reduction in SARS-CoV-2 viral load (2, 4). Two studies showed no significant differences in terms of viral load reduction [11,18].

#### 3.3.5 ß-cyclodextrin-citrox mouthwash

One study evaluated CDCM effectiveness: a significant reduction in viral load was described four hours after rinsing [9].

#### 3.3.6 Hypochlorous acid

One study evaluated the efficacy of HCIO which was previously approved as an effective disinfectant against COVID-19 with a dosage of 0.01%, whilst this study indicated that 0.02% HICO mouthwash had no significant efficacy regarding viral load reduction [19].

## 4 Discussion

The present systematic review investigated the efficacy of oral rinses against SARS-CoV-2. The eleven studies included a total of 1020 patients who were reviewed [[9], [10], [11], [12], [13], [14], [15], [16], [17], [18], [19]]. Since the emergence of SARS-CoV-2, global public health efforts have focused on preventative strategies such as vaccinations, social distancing, lockdowns, mask-wearing, and increased hand sanitizer use [14]. Some mouthwashes can inhibit the replication of SARS-CoV-2 by stopping its attachment to ACE2-positive epithelial cells [20] or can oxidize the structure of the virus [21,22].

CHX is a mouth rinse routinely used in dental offices for periodontal disease and to decrease infection rates in the oral cavity [23]. Administration of 0.12% CHX as both mouthwash and nasopharyngeal spray was found to have a high clearance of the SARS-CoV-2 viral load [13]. Elzian et al. found that using CHX rinse in patients for 30 seconds markedly reduced viral load [15]. Huang et al. highlight that CHX mouth rinse for 2-3 weeks can significantly prevent the spread to other individuals [13]. A study conducted by Fernanda et al. observed the use of CHX after rinsing with HP: Viral loads did not significantly reduce but by switching the order of mouthwash consumption an improvement can be recorded [10]. Bernstein et al. performed a study on the effectiveness of 0.12% CHX against herpes, influenza, and parainfluenza virus, and found a viral load reduction [24]. A systematic review by Fernandez et al. also found CHX to be an effective antiviral agent against HSV-1 and Influenza A viruses [25]. PI is an antiseptic mostly prescribed as a pre-and post-surgical disinfectant [26] against non-enveloped and enveloped viruses [22,27,28]. The studies included in this systematic review found significant viral load reductions when rinsing the oral cavity with a concentration of 0.5% and 1% PI [12,[14], [15], [16]]. However, when using 2% PI, no significant viral load reduction was seen [11]. Conflicting results were described by other studies: Lamas et al. found a noteworthy viral load reduction in SARS-CoV-2 [29], whereas Guenezan et al. reported PI to be ineffective in reducing nasopharyngeal viral load. PI usage also resulted in unwanted side effects such as nasal tingling and transient elevated TSH [30].

HP is an oxidizing agent with antiviral and antibacterial properties [31]. It works by disrupting the viral envelope and degrading the viral RNA [32]. A systematic review by Hossainian et al. sought to determine the effectiveness of hydrogen peroxide mouthwash on plaque and the gingiva. They found that short-term use had no significant impact on plaque reduction, but long-term use was correlated with benefits for the reduction of gingival inflammation (36). The studies included in our systematic review had varying results: two showed a significant decrease in the viral load [10,12], and one no decrease [11].

The antiviral effect of CPC is similar to PI. Both mechanisms destroy the lipid membrane of SARS-CoV-2, which could lead to a sustained impact on the salivary viral load reduction [14]. In the present systematic review, three studies on CPC were selected [10,11,14]. Two found a significant decrease in the viral load (2, 4), and one did not [11]. A randomized clinical trial by Mukherjee et al. evaluated the efficacy of CPC mouthwashes against coronavirus, influenza, and rhinoviruses: it did reduce the severity and duration of upper respiratory tract infections [33].

Only one study assessed the efficacy of CDCM in mild symptomatic and asymptomatic infected individuals four hours post-rinse [9].

Clinical application: administration of aforementioned mouthwashes can minimize the saliva SARS-CoV-2 viral load which can lead to a reduction of risk of spread of CPVID-19 which is a cost-effective strategy in reducing viral load both in dental settings and in the public.

This systematic review presents some limitations. There was a discrepancy in the populations of the studies included. Likewise, due to the limited raw data, we were not able to perform a meta-analysis of the included studies. The concentrations of the mouthwashes were not homogeneous in studies, and limited information about patients' oral conditions was reported. Our results must be interpreted with caution until further investigations are carried out.

In conclusion, this systematic review found that mouth rinses could effectively reduce the viral load of SARS-CoV-2. Also, this study indicated the efficacy of oral rinses in reducing the risk of transmitting the virus from SARS-CoV-2 positive patients, however, the results of some studies were contradictory, and due to the emergence of new SARS-CoV-2 variants and vaccinations, new scientific data are needed, as well as a shared methodology. Also, further studies should be conducted for a longer time as well as evaluating the preventive efficacy of oral rinses on negative SARS-Cov-2 individuals who do not develop the disease.

## Author's contribution

All authors have read and approved the manuscript.

## CRediT authorship contribution statement

**Pardis Ziaeefar:** Conceptualization, Investigation, Validation, Writing – original draft. **Narjes Bostanghadiri:** Data curation, Investigation, Validation, Writing – original draft. **Parsa Yousefzadeh:** Investigation. **Julian Gabbay:** Methodology, Writing – review & editing. **Amir Hashem Shahidi Bonjar:** Investigation. **Mitra Ghazizadeh Ahsaie:** Investigation. **Rosella Centis:** Methodology, Writing – review & editing. **Mohammad Sabeti:** Methodology, Supervision, Writing – review & editing. **Giovanni Sotgiu:** Methodology, Writing – review & editing. **Giovanni Battista Migliori:** Methodology, Writing – review & editing. **Mohammad Javad Nasiri:** Conceptualization, Investigation, Data curation, Methodology, Supervision, Validation, Writing – review & editing.

## Declaration of competing interest

The authors have no conflict of interests or any outside funding.
